# Monolayer nanocrystalline graphene synthesized from pyrolyzing a Langmuir monolayer of a polyaromatic hydrocarbon

**DOI:** 10.1126/sciadv.adv1856

**Published:** 2026-01-02

**Authors:** Xue Liu, Dario Calvani, Christopher Leist, Max Makurat, Adéla Melcrová, Meng He, Douwe Scholma, G. J. Agur Sevink, Francesco Buda, Yannick Hermans, Jan P. Hofmann, Haoyuan Qi, Xinliang Feng, Wouter H. Roos, Ute Kaiser, Grégory F. Schneider

**Affiliations:** ^1^ State Key Laboratory for Mechanical Behaviour of Materials, Xi’an Jiaotong University, Xi’an 710049, China.; ^2^ Leiden Institute of Chemistry, Faculty of Science, Leiden University, Leiden 2333 CC, Netherlands.; ^3^ Central Facility of Electron Microscopy, Electron Microscopy Group of Materials Science, Ulm University, Ulm 89081, Germany.; ^4^ Institute for Quantum Optics (IQO) and Centre for Integrated Quantum Science and Technology (IQST), Ulm University, Ulm 89081, Germany.; ^5^ Zernike Institute for Advanced Materials, Rijksuniversiteit Groningen, Groningen 9747 AG, Netherlands.; ^6^ Department of Biomaterials and Biomedical Technology, University Medical Center Groningen, A. Deusinglaan 1, Groningen 9713 AV, Netherlands.; ^7^ College of Materials Science and Engineering, Xi’an Shiyou University, Xi’an 710065, China.; ^8^ Leiden Institute of Physics, Faculty of Science, Leiden University, Leiden 2333 CC, Netherlands.; ^9^ Surface Science Laboratory, Department of Materials and Geosciences, Technical University of Darmstadt, Darmstadt 64287, Germany.; ^10^ Center for Advancing Electronics Dresden (cfaed) & Faculty of Chemistry and Food Chemistry, Technische Universität Dresden, Dresden 01062, Germany.

## Abstract

Bulk carbon materials can be synthesized through the modulation of precursor structures, stacking mode, and the optimization of pyrolysis conditions. The structure of two-dimensional (2D) carbon materials, however, is mainly driven by the pyrolysis temperature, pressure, and organic gas flow rates. Here, we report the synthesis of a centimeter-sized nanocrystalline graphene monolayer from pyrolyzing a Langmuir monolayer of a hexa(terpyridine)hexaphenylbenzene polyaromatic hydrocarbon (PAH) yielding a 2D carbon material with a thickness of 0.36 ± 0.07 nm. By designing the amphiphilic PAHs and reducing intermolecular π-π stacking interactions, a monolayer of horizontally aligned PAH was obtained at the water-air interface. During the pyrolysis, the monolayer served as a molecularly thin solid carbon source, leading to the controlled preparation of an electrically insulating nanocrystalline graphene monolayer. Our work proposes a 2D carbon material formed from the initial assembly of PAH monolayer at the water-air interface and subsequent pyrolysis and offers a modular strategy for designing nanocrystalline 2D films.

## Teaser

Nanocrystalline graphene is made from an aligned molecular monolayer at the interface.

## INTRODUCTION

Carbon materials are used in a wide range of applications, such as adsorption and membrane separation of gases and ions (*1*, *2*), energy storage devices (*3*), electronics (*4*), chemical sensors (*5*), and lighting (*6*), and they are usually prepared by pyrolysis of organic precursors. The engineering of bulk carbon materials is achieved by the modulation of precursors’ structure and stacking mode (*2*, *7*), and the optimization of pyrolysis conditions (*8*). However, for 2D carbon materials, such as graphene, amorphous carbon monolayer, and nanocrystalline graphene, the resulting film morphology is regulated mainly by pyrolysis conditions, for instance, temperature (*9*, *10*), pressure (*11*), and flow rates (*12*). Although many carbon precursors, such as methane, ethanol, and benzene, have been used for 2D carbon materials growth, modulation of the 2D carbon materials obtained by pyrolysis through the molecular structure of carbon precursors remained challenging because these processes use simple and volatile molecules, and the amount of carbon on the substrate cannot be controlled by the chemical structure of carbon precursors. Pristine graphene is a promising material due to its ultrathin nature and giant carrier mobility (*13*, *14*). Recent theoretical and experimental studies show that by reducing the grain size down to a few nanometers, nanocrystalline graphene/amorphous carbon monolayer with a large number of grain boundaries shows distinct electronic (*15*, *16*), mechanical (*17*, *18*), and thermal properties (*19*, *20*), which makes it an interesting material for fundamental research and technical applications. For instance, the nanocrystalline graphene-MoS_2_ van der Waals (VdW) heterostructure shows current rectified behaviors, and the photocurrent generated is about 500 times higher than that from the graphene-MoS_2_ VdW structure (*21*).

The research on approaches to obtain a nanocrystalline graphene monolayer with a certain degree of control of the internal monolayer morphology would benefit from a modular approach to structure these monolayers from the bottom-up. During the chemical vapor deposition (CVD) growth of nanocrystalline graphene, the high concentration of active carbon species provides high nucleation density and, therefore, the growth of multilayers, with an overall film thickness of more than a few nanometers (*22*–*24*). By quenching a high-temperature Pt foil in ethanol or by laser-assisted CVD growth on Cu substrate with carbon precursors, (*9*, *10*, *16*) well-defined nanocrystalline graphene was obtained. Self-assembled monolayers of aromatic thiols are also very efficient in synthesizing amorphous carbon films (*15*, *25*–*27*). Recently, we demonstrated the synthesis of a molecularly thin amorphous carbon monolayer (with a thickness of 2 nm) by pyrolyzing the Langmuir monolayer of amphiphilic hexa (2,2′-dipyridylamino)hexabenzocoronene polyaromatic hydrocarbon (PAH) (*28*, *29*). The carbon source for the amorphous carbon monolayer is provided by a nonvolatile, highly thermally stable PAH monolayer. The PAHs adopt an edge-on orientation on the surface of water and later on the substrate due to the π-π stacking effect and hydrogen bonds between water and the pyridine groups of the PAHs.

In this work, we demonstrate that by utilizing a PAH with a less-conjugated core and reducing intermolecular π-π stacking interactions, the PAH now horizontally aligns in its monolayer, which can be converted into a monolayer nanocrystalline graphene by pyrolyzation with a resulting monoatomic thickness. The densely packed PAH monolayer offers high-density nucleation centers and a limited amount of solid carbon source. The large molecule weight prevents the sublimation before cross-linking. The horizontally aligned PAHs serve as an atomic thin carbon source and can substantially reduce the nanocrystalline graphene thickness down to one carbon thick. The direct growth of nanocrystal graphene monolayer on noncatalytic substrates such as SiO_2_/Si wafers prevents postgrowth transfer, and enables a clean and cracks free surface ready for direct device integration or use as a free-standing membrane. TEM results show that the structure of nanocrystalline graphene contains small crystalline grains that are randomly oriented and lack long-range order. The nanocrystalline graphene thus prepared is nonconductive and exhibits at least 100 times lower stability than graphene upon laser exposure. Atomic force microscopy (AFM) nanoindentation experiment shows that the nanocrystalline graphene has good mechanical properties with a penetration force of 70 ± 20 nN.

## RESULTS

### Synthesis of nanocrystalline graphene

**Fig. 1.**
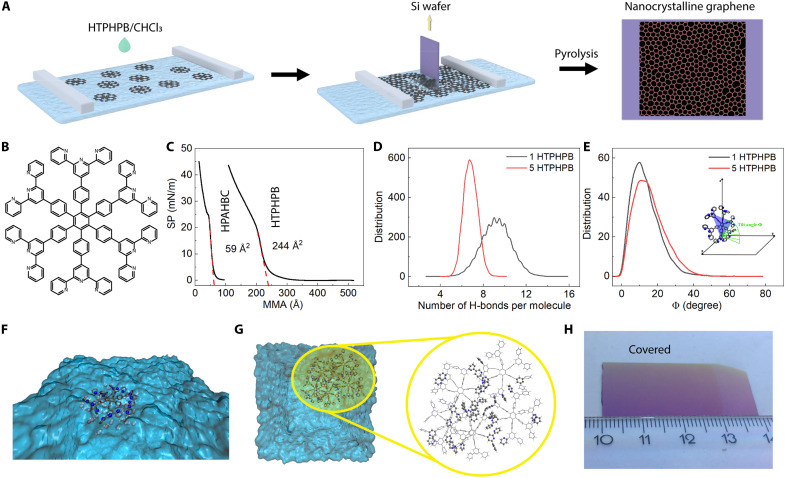
2D nanocrystalline graphene preparation. (**A**) Scheme illustrating the 2D nanocrystalline graphene monolayer preparation. (**B**) Chemical structure of HTPHPB. (**C**) Compression isotherm of HTPHPB on water compared to the compression isotherm of HPAHBC. The mean molecular area (MMA) is about 244 Å^2^, close to the HTPHPB molecular size derived from its single-crystal structure. (**D**) Distribution of the number of hydrogen bonds (H-bonds) per HTPHPB molecule from MD simulations with one and five HTPHPB molecules. The Gromacs Tool gmx hbond was used for the calculation of the number of H-bonds determined on the basis of cutoffs for the angle H_water_-O_water_-N_HTPHPB_ of 35° and the distance O_water_-N_HTPHPB_ of 3.5 Å. OH is regarded as the donor, and N as the acceptor. (**E**) Distribution of tilt angles (Φ) for each molecule from MD simulations with one and five HTPHPB molecules. Inset: illustration of the tilt angle Φ (green arc) between the blue plane defined by three specific nitrogen atoms on the edge and the *x*-*y* plane; carbon atoms in gray, nitrogen atoms in blue, and hydrogen atoms omitted for clarity. (**F**) A representative snapshot of one HTPHPB on the water surface with first solvation shell water molecules. For HTPHPB, carbon atoms are in gray, nitrogen atoms in blue, and hydrogen atoms omitted for clarity. For water, oxygen and hydrogens atoms are in red and white, respectively. (**G**) A representative snapshot from the MD simulation includes five HTPHPB molecules on the water surface, showing the most frequent orientation and packing adopted by the molecules. We highlight in the yellow circle the five molecules planar cluster with 42 interacting pyridines in the central X-shape core and in the external pairs involved in the π-π stacking interactions. The π-π stacking interacting moieties are depicted in balls and sticks, while the rest of the molecules are only shown with sticks. Carbon atoms are in gray, nitrogen atoms in blue, and hydrogen atoms in white. (**H**) Digital photo of nanocrystalline graphene prepared on Si/SiO_2_ wafer (about 3.5 × 2 cm^2^).

The centimeter-sized nanocrystalline graphene monolayer was prepared by pyrolyzing the Langmuir monolayer of hexa(terpyridine)hexaphenylbenzene (HTPHPB) monomer (Fig. 1, A and B) under an inert atmosphere. The monomer HTPHPB was synthesized following the literature procedure (figs. S1 and S2) (*30*, *31*). A chloroform solution of HTPHPB (1 mg/ml) was spread on the water surface in a Langmuir-Blodgett trough. After the full evaporation of the chloroform (30 min after depositing the HTPHPB solution), the barrier was closed slowly at a speed of 1 mm/min until the desired surface pressure of 15 mN/mm was reached. The mean molecule area (MMA) obtained from the isotherm curve is about 244 Å^2^ (Fig. 1C), which is close to the size of HTPHPB calculated from its single crystal structure (fig. S3), indicating that the monomers adopted a face-on configuration on the water surface.

The behavior of the HTPHPB molecules on the water surface was further explored by molecular dynamics (MD) simulations. A water slab has been considered with different amounts of HTPHPB molecules adsorbed on both water surfaces (see the section “Simulations” in Materials and Methods for more information). These MD simulations highlight the predominant role of the hydrogen bonds (H-bonds) between the water molecules and the HTPHPB guiding the arrangement at the interface. This is in contrast with a previous work on the water–hexa(2,2′-dipyridylamino)hexabenzocoronene (HPAHBC) interface (*28*), where we observed competition between π-π intermolecular stacking interactions between large coronene cores and hydrogen bond interactions of the molecules on the water surface. A single HTPHPB molecule isolated on the water surface leads to an average of nine hydrogen bonds (Fig. 1D, black curve). The molecule is nearly flat on the water surface with a tilt angle of ~13° (Fig. 1, E, black curve, and F). When considering five HTPHPB molecules randomly distributed on the water surface, the simulations show that the molecules lie essentially parallel to the water surface with an average of seven hydrogen bonds (Fig. 1D, red curve) and tilt angle ~15.5° (Fig. 1E, red curve, and G). The small difference in the hydrogen bond amount between one and five HTPHPB molecules can be explained by the presence of pyridine interactions in the latter. On average, the thickness of the five-molecule cluster is around 9.5 Å, in good agreement with the experimental value after Langmuir-Blodgett compression (7 Å, fig. S4). Figure 1G shows these five-molecule cluster from the top in a representative snapshot during the last 10 ns of the MD production run. Zooming on the cluster, we highlight in balls and sticks 42 pyridines that play a role in the π-π stacking interactions. The average distance between pyridines in dimer or trimer groups is ~3.6 Å with either parallel-sandwich, antiparallel-sandwich, parallel-displaced, antiparallel-displaced, or T-shaped configurations (*32*, *33*). We note a central X-shape core of interaction that strengthened and stabilized symmetrically the cluster, which might represent the seed of the nanocrystal graphene formation (Fig. 1G). Calculations at density functional tight binding (DFTB) with D3BJ dispersion correction level on the five HTPHPB molecules cluster and one HTPHPB molecule have been performed. On the basis of these calculations, the estimated π-π stacking interaction energy per interacting pyridine is around −3.30 kcal mol^−1^, which is in good accordance with the literature (table S1) (*32*). The hydrogen bond energy between pyridine and water is about 3.70 kcal mol^−1^, which is comparable to the π-π stacking energy between pyridines (*34*). The pyridine-stacking of five molecules leads to a reduction of approximately two hydrogen bonds per HTPHPB molecule, corresponding to a total energy loss of around 37 kcal mol^−1^. However, the π-π stacking between 42 interacting pyridines within five molecules produces an energy gain of −138.47 kcal mol^−1^ (table S1), thereby stabilizing the HTPHPB molecules in a planar pyridine-stacked configuration at the interface with water (Fig. 1G).

**Fig. 2.**
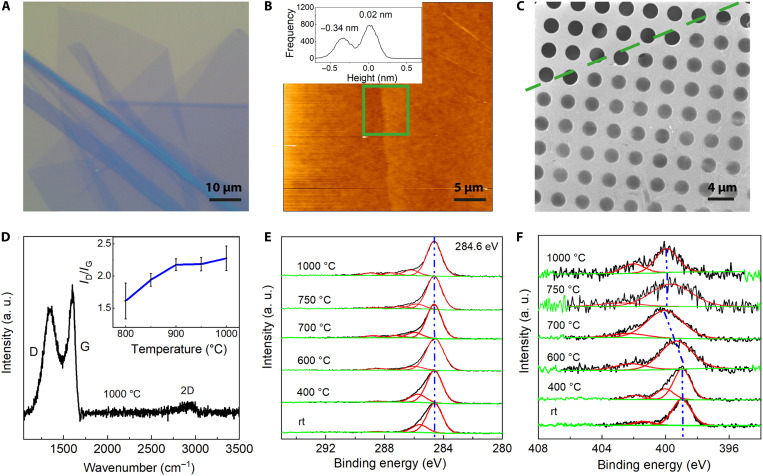
Characterization of the 2D nanocrystalline graphene. (**A**) Optical image of 2D nanocrystalline graphene monolayer transferred on a Si/SiO_2_ wafer. (**B**) AFM image of a 2D nanocrystalline graphene monolayer on a Si/SiO_2_ wafer. The thickness of the monolayer is about 0.36 nm, with an average roughness (Ra) of 0.07 nm. (**C**) SEM image of the nanocrystalline graphene transferred onto the Quantifoil grid with holes of 2 μm. The monolayer is mechanically strong enough to be free-standing over 2-μm holes. (**D**) Raman spectrum of nanocrystalline graphene monolayer annealed at 1000°C. Insert, the intensity ratio between D and G peaks for the nanocrystalline graphene prepared from 800° to 1000°C. (**E**) and (**F**) C 1s and N 1s X-ray photoelectron spectra of monolayer annealed at different temperatures.

Later, the Langmuir monolayer was transferred onto a Si wafer by vertical deposition. The HTPHPB monolayer before annealing is intact without cracks and can be free-standing, probably due to the interactions between pyridines from adjacent HTPHPB monomers (figs. S5 and S6). The circle pattern in the AFM image may originate from the vertical transfer process (figs. S4 and S7), which seems to have no influence on the nanocrystalline graphene quality. Before the transfer, the Si wafer was treated with O_2_ plasma (200 W) for 1 min to form a hydrophilic surface. The Si wafer with HTPHPB monolayer on top was then annealed for 15 min in vacuum (1 mbar, Ar atmosphere). The nanocrystalline graphene monolayer on Si/SiO_2_ wafer gives similar contrast as monolayer graphene (Figs. 1H and 2A and fig. S8). The thickness of the nanocrystalline graphene monolayer is 0.36 nm with an average roughness (Ra) of 0.07 nm (Fig. 2B), which shares the same thickness as single-layer graphene and is thinner than an amorphous carbon monolayer (*9*), indicating the atomic thin nature of the nanocrystalline graphene. The scanning electron microscopy (SEM) image of the nanocrystalline graphene on the Quantifoil grid shows that the monolayer has good mechanical strength and can be free-standing over at least 2-μm openings (Fig. 2C).

### Structural characterization of nanocrystalline graphene

The annealing temperature needs to be sufficiently high to trigger the rearrangement of carbon atoms. Raman spectroscopy was carried out to characterize the membrane after different annealing temperatures (fig. S9). Because of the absence of large-scale conjugation, the HTPHPB monolayer exhibits no Raman signal. After annealing above 700°C, intense D and G peaks were observed, corresponding to the bond stretching of sp^3^-hybridized defects and sp^2^-bonded pairs (*35*). Note that only weak 2D peaks can be found, suggesting no long-range order even at annealing temperatures up to 1000°C (Fig. 2D). Since samples annealed below 700°C did not give rise to D and G peaks, we conclude that the conjugation structure first emerged at ca. 700°C, which is in perfect agreement with the transition temperature determined by x-ray photoelectron spectroscopies (fig. S10). The intensity ratio between the D and G peaks (*I*_D_/*I*_G_) increased from ca. 1.61 at 800°C and 2.27 at 1000°C (Fig. 2D, insert), which indicates an average defect distance *L*_D_ of around 1.5 nm (*36*, *37*).

The annealing process under various temperatures was further probed via x-ray photoelectron spectroscopy (XPS). Before annealing, the C 1s XPS spectrum of HTPHPB can be described by two main components, corresponding to sp^2^ carbon at 284.6 eV and C-N at 285.7 eV (Fig. 2E). The N 1s XPS spectrum of HTPHPB monomer contains a main peak at 398.9 eV, corresponding to pyridinic N atoms (Fig. 2F). By increasing the annealing temperature (up to 1000°C), the C 1s spectrum changes slightly to be dominated by sp^2^ carbon. However, the N 1s main peak broadens with increasing annealing temperature, shifting to 400.0 eV at 600°C. A further upshift in binding energy occurs at 700°C, which can be ascribed to tertiary nitrogen. Upon annealing above 700°C, the N 1s signal weakens significantly in intensity, indicating decomposition of the pyridine motifs (fig. S10). The monolayer annealed above 700°C can be free-standing, further supporting that the atomic rearrangement occurs at ca. 700°C (fig. S11).

**Fig. 3.**
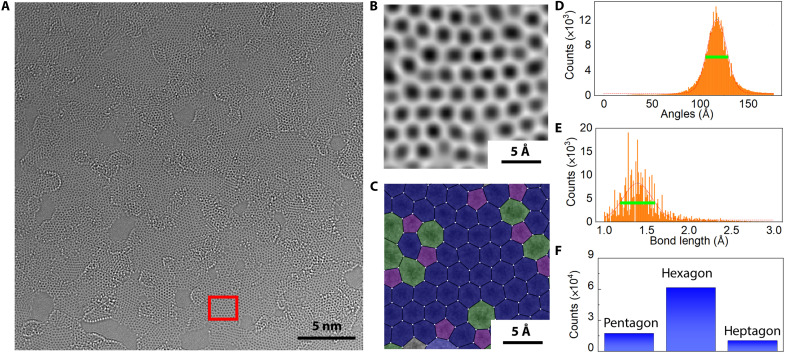
AC-HRTEM characterization of nanocrystalline graphene monolayer pyrolyzed at 1000°C. (**A**) AC-HRTEM image of the nanocrystalline graphene monolayer. Carbon atoms appear bright on a dark background. (**B**) Magnified image from the boxed region in (A). Minimum filtering has been applied for better visibility of the polygons. (**C**) Atomic positions (white) and polygons (colored) mapped by neural network analysis. Pentagon: purple. Hexagon: blue. Heptagon: green. (**D**) Statistical analysis of bond angle distribution for nanocrystalline graphene monolayer (number of bond angles considered: 6 × 10^6^). The FWHM of bond angles in nanocrystalline graphene (24.0°) is broader than that of CVD graphene (16.3°). (**E**) Statistical analysis of bond angle distribution for nanocrystalline graphene monolayer (number of bond lengths considered: 4 × 10^6^). The bond length of nanocrystalline graphene (0.39 Å) exhibits broadening compared to CVD graphene (0.27 Å). (**F**) Frequency of polygons (number of polygons considered: 9 × 10^5^).

Despite the spectroscopic characterizations providing macroscopic information, the local structure of the annealed monolayers was further explored at the atomic level by using aberration-corrected high-resolution transmission electron microscopy (AC-HRTEM). Selected-area electron diffraction exhibits two rings at 4.7 and 8.1 nm^−1^, coinciding with graphitic carbon (fig. S12). The diffusive nature of the diffraction rings demonstrates the lack of long-range order, which is in agreement with Raman spectroscopy. Figure 3A presents a typical AC-HRTEM image of the nanocrystalline graphene monolayer annealed under 1000°C, showing nanometer-scale graphene patches embedded in a defective matrix (Fig. 3B). Because of the chromatic and spherical aberration corrections, the instrumental resolution is down to 80 pm when operating under 80 kV. Therefore, the carbon atoms can be unambiguously resolved, allowing for straightforward structural determination. However, because of the disordered atomic arrangement, quantitative structural analysis (e.g., determining bond lengths and angles, the occurrence frequency of polygons) is not a trivial task. Therefore, we have developed a neural network based on U-net architecture to automatically determine atomic positions in AC-HRTEM images (see Materials and Methods) (*38*). By determining the position of every single atom, the distribution of bond length and angle, and polygon percentage can be extracted statistically. For instance, Fig. 3C illustrates the polygon mapping based on our algorithm. To enhance the accuracy of the statistical analysis, we have incorporated segmentations into the neural network, such that nonmonolayer regions induced by surface contamination and local thickness variation are excluded. By evaluating 34 experimental AC-HRTEM images with 29 nm × 29 nm field of view, we found that nanocrystalline graphene (Fig. 3, D and F) exhibits a substantial broadening of the bond length and angle distributions as compared to CVD graphene (fig. S13). The full width at half maximum (FWHM) for bond angle distribution increased from 16.3° (CVD graphene) to 24.0° (nanocrystalline graphene), and the FWHM of the bond length distribution peak is broadened from 0.27 (CVD) and 0.39 Å (nanocrystalline), whereas the peaks are still centered at 120° and 1.4 Å, manifesting the presence of sp^2^ carbon with short-range order. Figure 3F demonstrates the statistics of polygon frequency derived by real-space mapping. When comparing with the statistics of CVD graphene (fig. S13), we observed a substantial increase in the pentagons at the cost of hexagons, whereas the percentage of heptagons increased only slightly (table S2). Figures S14 and S15 present the comparison between nanocrystalline graphene annealed under 750° and 1000°C. As the annealing temperature increased, a higher fraction of monolayer regions (sp^2^) can be observed, and the grain size is around 2 nm, a similar phenomenon was observed in the experiment of in situ annealing of nanocrystalline graphene in TEM (fig. S16). These results suggest that, although annealing above 700°C could already fully decompose the pyridine motifs and trigger atomic rearrangement, the remaining carbon atoms from the HTPHPB molecules first adopt a highly disordered configuration at the early stage, serving as an ultrathin carbon reservoir. Further energy transfer via heating drives the carbon atoms into an energetically favorable crystalline state, leading to the formation of nanocrystalline graphene monolayers.

### Studies of conductivity and mechanical properties

**Fig. 4.**
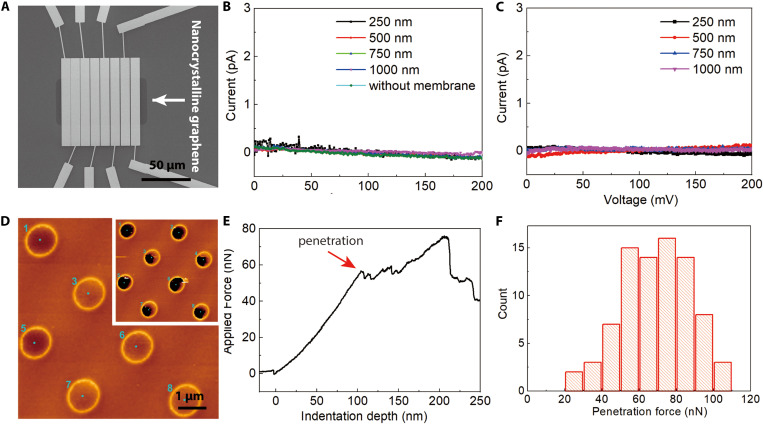
Conductivity and mechanical properties of nanocrystalline graphene. (**A**) SEM micrograph of the device used for conductivity measurements. The nanocrystalline graphene was prepared on a Si wafer, and the electrodes were placed on top by lithography. The details about the design are shown in fig. S17. (**B**) *I-V* plots measured at room temperature for a nanocrystalline graphene monolayer prepared at 750° and (**C**), 1000°C. The results show that the prepared nanocrystalline graphene is an insulator. The distances between the electrodes are 250 nm, 500 nm, 750 nm, and 1 μm respectively. (**D**) AFM point indentations of the nanocrystalline graphene over 1.2-μm holes. Insert, after the indentations. Green dots show the points of indentation. (**E**) An example of the force indentation curve from one of the spots depicted in Fig. 1D. In all cases, the film broke at forces <120 nN, and we see a penetration event in the force indentation curve as a sudden drop in the applied force. (**F**) Detailed histogram of the penetration forces measured for the nanocrystalline graphene sample.

As the nanocrystalline graphene monolayer is prepared on an insulating substrate (Si wafer), it is possible to measure the resistance directly (Fig. 4A and fig. S17). Unlike graphene, which shows high electron mobility and low resistance, the monocrystalline graphene monolayers prepared at 750° and 1000°C are insulating (Fig. 4, B and C). The current under 200 mV is below 1 pA and noisy. The resistance value is above 10 TΩ sq.^−1^, and it is the highest number reported so far among 2D carbon membranes. (*9*, *16*) This nonconductive nature, compared to the monolayer amorphous carbon films and nanocrystalline graphene reported in the literature (*9*, *10*), is likely attributable to the inert Si wafer substrate used during its preparation, and a high density of defects cannot be effectively repaired during high-temperature growth. As can be seen from fig. S18, the monolayer prepared at 750°C is fully etched after about 6 seconds of exposure to the blue laser (457 nm, 2 mW, laser spot diameter of around 2 μm). In comparison, there are no detectable changes with pristine graphene even after 10-min of laser exposure (457 nm, 2 mW; fig. S19). The nanocrystalline graphene prepared at 1000°C shows, however, slightly better structural stability upon laser exposure (fig. S18A). The etching process is due to thermal oxidation and sublimation (*39*). The stability difference between nanocrystalline graphene and pristine graphene can be due to the abundance of grain boundaries and small grain size, which largely decreases the thermal conductivity (*40*). We demonstrate the possibility of 2D patterning of nanocrystalline graphene by laser scanning. Figure S18A shows the real-time patterning process of nanocrystalline graphene prepared at 1000°C. The nanocrystalline graphene nanopattern is fabricated directly on a Si/SiO_2_ wafer; no transfer step can be a benefit for applications in nanoelectronics. These properties make nanocrystalline graphene an interesting material for laser processing of micro/nanopatterns in 2D materials for (micro)electronics (*39*).

The mechanical properties of nanocrystalline graphene were studied by a combined AFM imaging and nanoindentation approach (Fig. 4D and figs. S20 and S21) (*41*). The center of the freestanding nanocrystalline graphene over 1.2-μm holes (UltrAufoil) was indented with a tip on the cantilever until failure. A force-indentation curve was recorded as shown in Fig. 4E. The nanocrystalline graphene fails at an average point penetration force of 70 ± 20 nN. In pristine graphene, the maximum indenting force of 700 nN was not always sufficient to penetrate. A comparison of the indentation experiments on nanocrystalline and pristine graphene is provided in figs. S20 and S21. Compared to graphene, its inferior mechanical strength likely originates from structural defects and cracks (fig. S22).

## DISCUSSION

We have successfully demonstrated the possibility of regulating the thickness of 2D carbon materials by using HTPHPB with a less conjugated core compared to HPAHBC. By the synergistic effect of H-bonding between water and pyridines, and π-π stacking interactions between pyridines of neighboring molecules, HTPHPB molecules are being oriented flat on the surface of water. The chemical structure and properties of the nanocrystalline graphene were further studied, showing short-range crystallinity of the film, an electrically insulating behavior, a poor stability upon laser exposure, which may be useful for lithography, and importantly, an atomic thin nature, making it potentially useful as a free-standing membrane and in nanoelectronics.

## MATERIALS AND METHODS

### Materials

All chemicals were purchased commercially and used without purification. HTPHPB was synthesized following literature procedures and purified by column chromatography over Al_2_O_3_ using DCM and MeOH (*30*). Fourier transform infrared (FTIR) was recorded on a Perkin-Elmer Paragon 1000 FTIR. Langmuir film was prepared on a Langmuir-Blodgett trough (KSV NIMA, Finland). Annealing was performed in a nanoCVD (Moorfield). The optical image was recorded on a Leica DM 2700M microscope. AFM images of the layers on Si/SiO_2_ wafers were recorded with an JPK NanoWizard Ultra Speed machine by an intermittent contact mode (tapping mode) in air at room temperature. The AFM-based mechanical measurements were performed with a JPK Nano Wizard Ultra Speed machine (see below at AFM nanoindentation experiments). SEM images were recorded on a FEI NOVA SEM. Raman spectroscopy was performed on a WITec confocal spectrometer using a 532-nm laser. Conductivity was measured with a patch clamp amplifier (Axopatch 200B).

### Simulations

Geometry preoptimization and then optimization of the HTPHPB molecule in the gas phase were performed respectively using UFF and density functional theory (DFT) at B3LYP-D3 level, dispersion correction by Grimme *et al.* (*42*), and the TZP basis set, with ADF2019 (*43*). All MD simulations were carried out using GROMACS 2016 (*44*–*50*), and visual molecular dynamics (VMD) (*51*) was used for visualization. The particle mesh Ewald method was employed to accurately compute electrostatic interactions (*52*). The cutoff for the Coulomb interaction and Lennard-Jones interaction was set at 10 Å. During the canonical NVT (with constant number of particles N, volume V, and temperature T) simulation, the temperature was kept fixed with the V-rescale coupling method (*53*). DFTB single points for a cluster of five HTPHPB molecules and single HTPHPB molecule geometries, both extracted from the last snapshot of the last 10 ns of MD, were performed in the gas phase using DFTB-D3BJ with the 3ob-3-1 parameters (DFTB.org) and using ADF2019 (*54*, *55*).

### Simulation settings for water interface

A simulation of a water slab containing 32985 water molecules centered in a periodic box (10.0 × 10.0 × 20.0 nm^3^) has been carried out to establish a water model using the TIP4P-Ew/2004 force field (*56*). The system was first energetically minimized and then was equilibrated for 8 ns with NVT at 300 K.

### Simulation settings for the water/HTPHPB interfaces

In the MD simulation of HTPHPB molecules on water, the organic molecules were simulated using the OPLS-AA force field (*57*–*59*) and PRODRG (*60*) parameterization (see table S3) with the charges calculated with RESP model (*61*) using GAUSSIAN 16 (*62*) and Amber Tools (*63*) on the previously DFT optimized structure of the HTPHPB molecule. We packed in our water box using PACKMOL18 program (*64*, *65*), first one, and then five of previously optimized HTPHPB molecules, respectively. An equal number of HTPHPB molecules were placed on both sides of the water slab producing two HTPHPB/water interfaces. We could improve the statistics on our results by averaging on both interfaces, which can be considered independent due to the thickness of the water box and the vacuum space above each interface along the *z* axis. We initialized the simulations with a different number of HTPHPB molecules, one and five, on each side of the slab, respectively. For each case, the molecules were initially arranged in a planar starting configuration. Each system was first equilibrated with NVT at 70 K for 5 ns, and then the temperature increased to 300 K for the other 5 ns. After these preequilibration simulations, we ran a final NVT simulation at 300 K for 20 ns, of which the final 10 ns was used for our data production and analysis (*66*).

### X-ray photoelectron spectroscopy

X-ray photoelectron spectroscopy was used for surface analysis and was carried out within the DArmstadt Integrated SYstem for FUNdamental research (DAISY-FUN), using a SPECS Phoibos 150 analyzer (SPECS GmbH, Berlin, Germany) and a SPECS Focus 500 X-ray source using the monochromatized Al K_α_ line at 1486.74 eV. Binding energies were calibrated by adjusting to the Ag 3d_5/2_-core level (*E*_B_ = 368.3 eV), Cu 2p_3/2_-core level (*E*_B_ = 932.7 eV), and Pt 4f_7/2_-core level (*E*_B_ = 71.2 eV) from freshly sputter-cleaned samples.

### Aberration-corrected high-resolution transmission electron microscopy

AC-HRTEM images were acquired with the image-side *C*_C_/*C*_S_-corrected SALVE instrument operated under 80 kV with a point resolution of <0.08 nm. The selected-area electron diffraction pattern was acquired with a Thermo Fisher Scientific Talos 200X operated under 80 kV.

### Neural network of U-net type for automatic evaluation of nanocrystalline graphene monolayers.

#### Data for training

Simulated TEM images were used for the training of the neural network. The use of simulated images eliminates the need for hand labelling, thus avoiding selection bias and significantly reducing time cost. The atomic models were created with the atomic simulation environment (*67*). HRTEM image simulations of the created models are carried out using abTEM (*68*). The modelling of the semi-random carbon monolayer was created with the method described in the literature (*69*). In this method, randomly distributed seed points are generated. A Voronoi Tesselation is carried out with these seed points. Carbon atoms are placed at the resulting vertices. For each image, four masks are created: the atom positions, the positions of polygon centers (i.e., the position of the seed points), holes in the carbon membrane, and segmentation for the nonmonolayer region. For nonmonolayer segmentation, a continuous layer of graphene is added to the randomized carbon model. The corresponding masks differentiate the monolayer and nonmonolayer areas. The images are simulated on the basis of experiment parameters (i.e., pixel size, defocus, aberrations, electron dose, etc.). A linear image gradient of random direction and strength is added to the simulated images. This image gradient markedly improved the robustness of the network against intensity variations in the image background. The image size of the training images was 512 × 512 pixels for segmentation of monolayer and vacuum areas and 256 × 256 pixels for atom and center position with a pixel size corresponding to the that in the experimental TEM images, a defocus range 10 to 50 Å and an electron dose rate of (1 − 3) × 10^5^ e^−^/Å^2^.

#### Training

A neural network of a modified U-net type was trained with training sets of between 1000 and 4000 images (*38*, *69*). Four separate networks were trained for the four masks: the atom positions, the positions of polygon centers (i.e., the position of the seed points), holes in the carbon membrane, and segmentation for the nonmonolayer region. The network was trained with an NVIDIA GeForce RTX 3060 graphics card with 12 GB VRAM.

#### Appling the network on experimental TEM images

Depending on the imaging conditions and sample thickness, the image contrast can vary substantially. Because the training is carried out on small images which contain up to two carbon layers, the contrast range in the training images is smaller than the experimental images. Therefore, the experimental TEM images are normalized to 1 and multiplied to fit the training conditions to compensate for this issue.

#### Processing the neural network’s output

A binary map is applied to the output of the neural network, from which the atom and polygon center positions are extracted. To convert the neural network output into atom positions, we used ndimage.label in conjunction with ndimage.find_objects applied to the difference between the maximum and minimum filtered images. The program iterated through the found objects and determined their centers. The positions were then saved in a list. To correctly identify C─C bonds, a Delaunay triangulation between the atoms and center positions is used. The bonds are isolated by subsequential removal of the connections to the center points, allowing the determination of bond lengths and angles. The statistical analysis is plotted in a histogram with binning 200, and a Gaussian fitting has been applied to determine the FWHM. Meanwhile, the center positions and their connections to nearby atoms describe the carbon polygons for the polygon mapping.

#### AFM nanoindentation experiments

Graphene or nanocrystalline graphene were transferred on to a TEM grid (Au deposited, pore diameter of 1.2 μm) by poly(methyl methacrylate) transfer method. AFM tips from Bruker, TESP-V2 (TESPA-V2), were used with a nominal tip radius of 7 nm. All experiments were performed in air using a JPK Nano Wizard Ultra Speed AFM. Graphene or nanocrystalline graphene was imaged in QI imaging mode. Holes fully covered with the film were identified by measuring the height profile along the cross sections over the middle of the covered holes. Next, the AFM was switched from imaging mode to force spectroscopy mode. A single point at the center of the fully covered hole was selected for the point indentation. Indentation was performed up to the maximum applied force of 700 nN. Higher forces do not always penetrate the graphene film, but they damage the silicon tip of the TESPA-V2 cantilevers. The loading rate of the cantilever moving down toward the sample and pushing on it was 300 nm/s and during this movement a force-distance curve was recorded. If there were multiple fully covered holes in the field of view, they were all indented with the same maximum force and loading rate. When there is a significant dip in the applied force, namely, a drop >2 nN, we identify this event as the penetration. After the indentation experiments, the machine was switched back to the QI imaging mode and the same region was imaged after the indentations to see whether the films had ruptured or stayed intact.
