# Supplementary material for: Monolayer nanocrystalline graphene synthesized from pyrolyzing a Langmuir monolayer of a polyaromatic hydrocarbon
Source: Sci Adv. 2026 Jan 2;12(1):eadv1856. doi: 10.1126/sciadv.adv1856 (PMC12758557; doi:10.1126/sciadv.adv1856)
Supplement: Supplementary file 1 — Figs. S1 to S22 Tables S1 to S3 [file sciadv.adv1856_sm.pdf]

Science Advances
AAAS

Supplementary Materials for

Monolayer nanocrystalline graphene synthesized from pyrolyzing a Langmuir monolayer of a polyaromatic hydrocarbon

Xue Liu *et al.*

Corresponding author: Xue Liu, liuxue@xjtu.edu.cn; Grégory F. Schneider, g.f.schneider@chem.leidenuniv.nl

Sci. Adv. **12**, eadv1856 (2026)
DOI: 10.1126/sciadv.adv1856

This PDF file includes:

Figs. S1 to S22
Tables S1 to S3

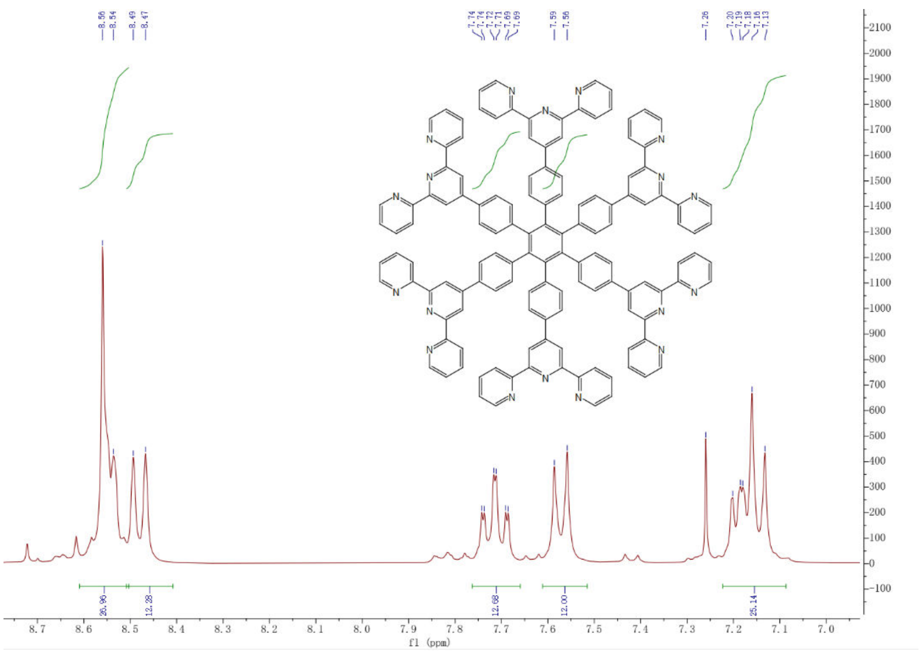

Fig. S1. ^{1}H NMR spectrum of HTPHPB ($CDCl_3$).

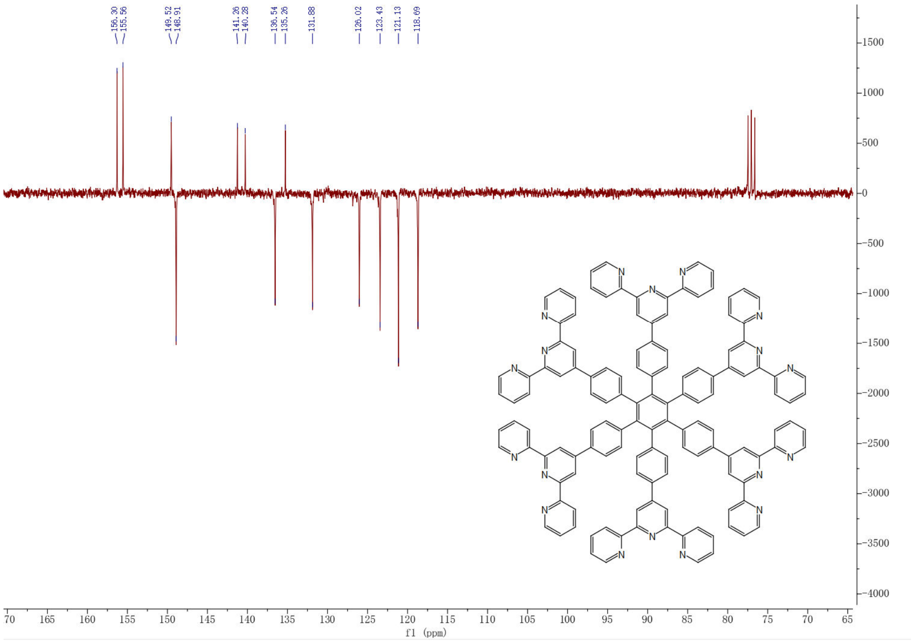

Fig. S2. ^{13}C NMR spectrum of HTPHPB ($CDCl_3$).

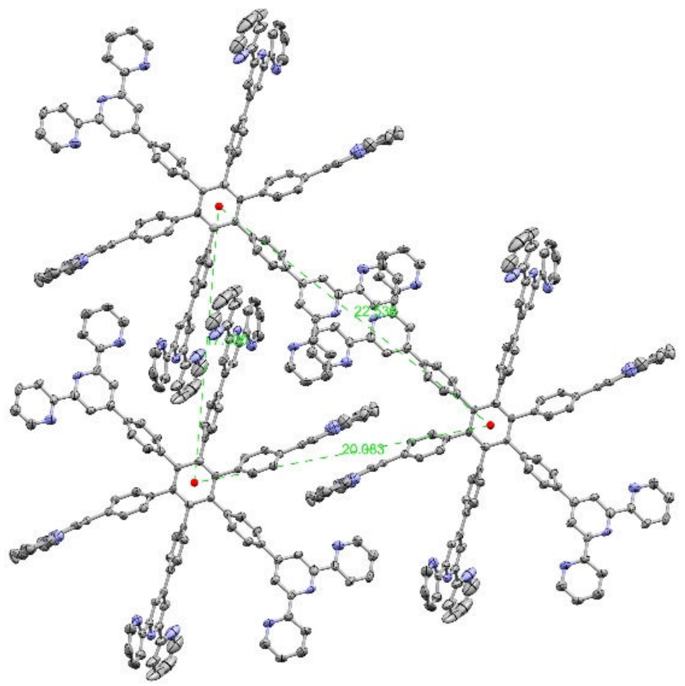

Fig. S3. Packing mode and distance between HTPHPB in its single-crystal structure.

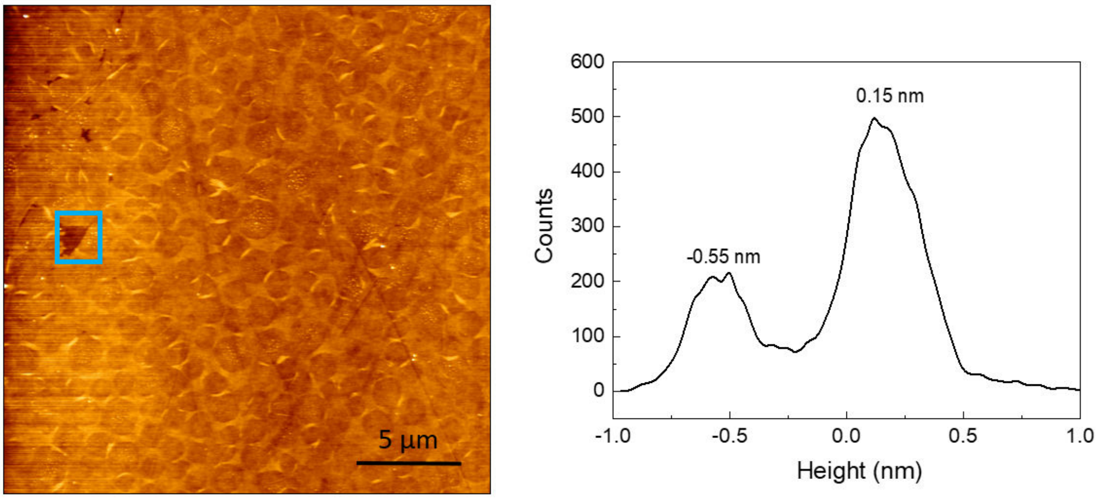

Fig. S4. AFM image of HTPHPB LB membrane before annealing. The thickness of the membrane is about 0.7 nm.

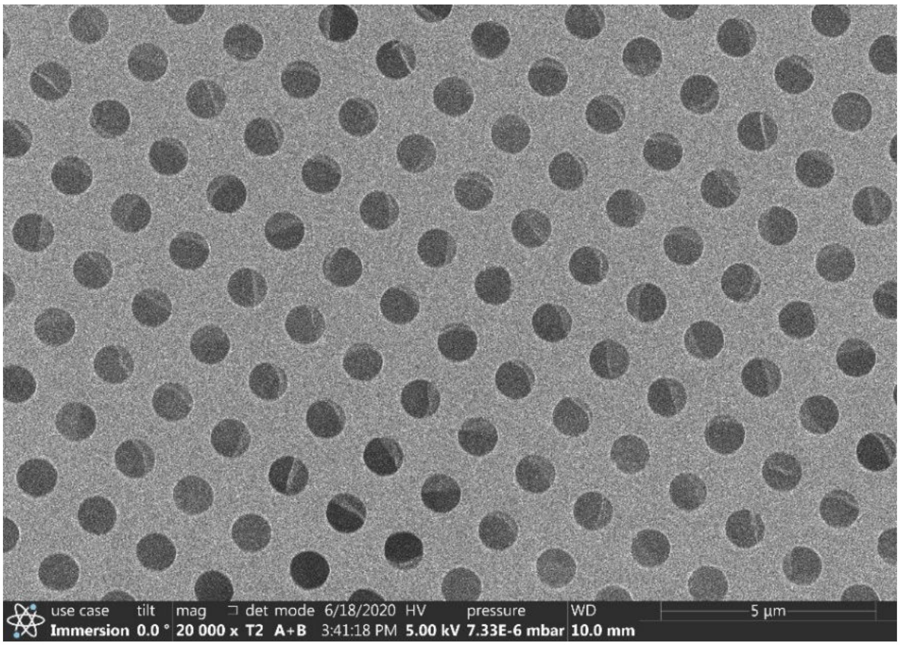

Fig. S5. SEM image of HTPHPB Langmuir monolayer directly transferred from Langmuir trough without annealing.

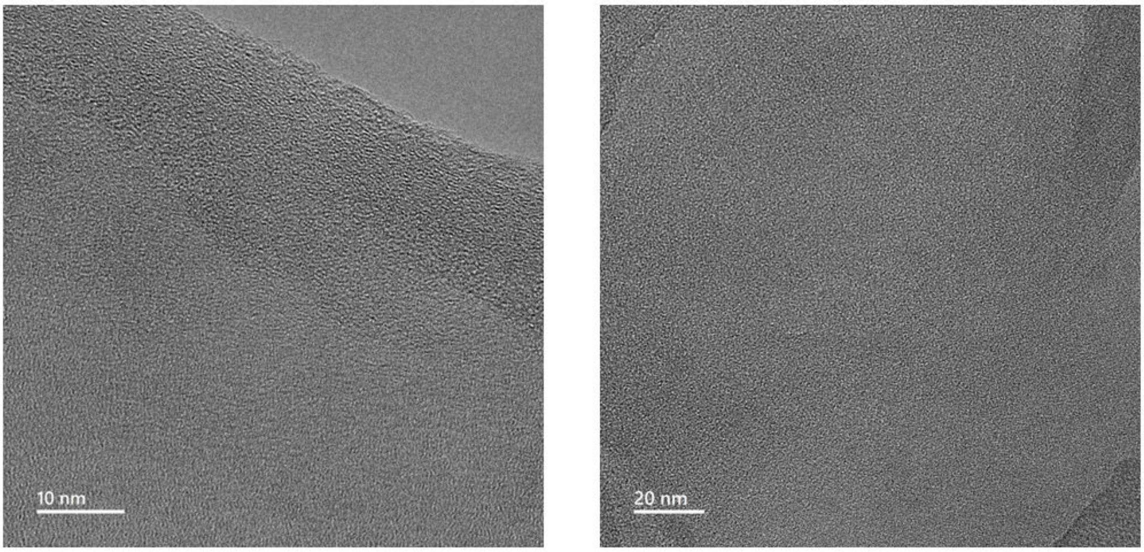

Fig. S6. TEM images of HTPHPB monolayer on TEM grid before annealing transferred by Langmuir Blodgett method (vertical transfer).

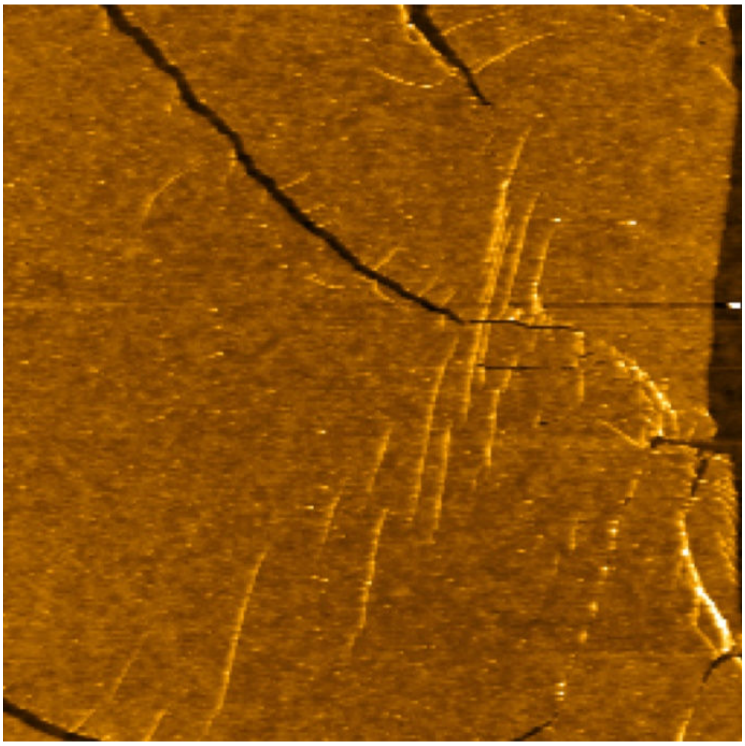

Fig. S7. AFM image of HTPHPB monolayer transferred on Si wafer by Langmuir Schaefer method (horizontal transfer) before annealing.

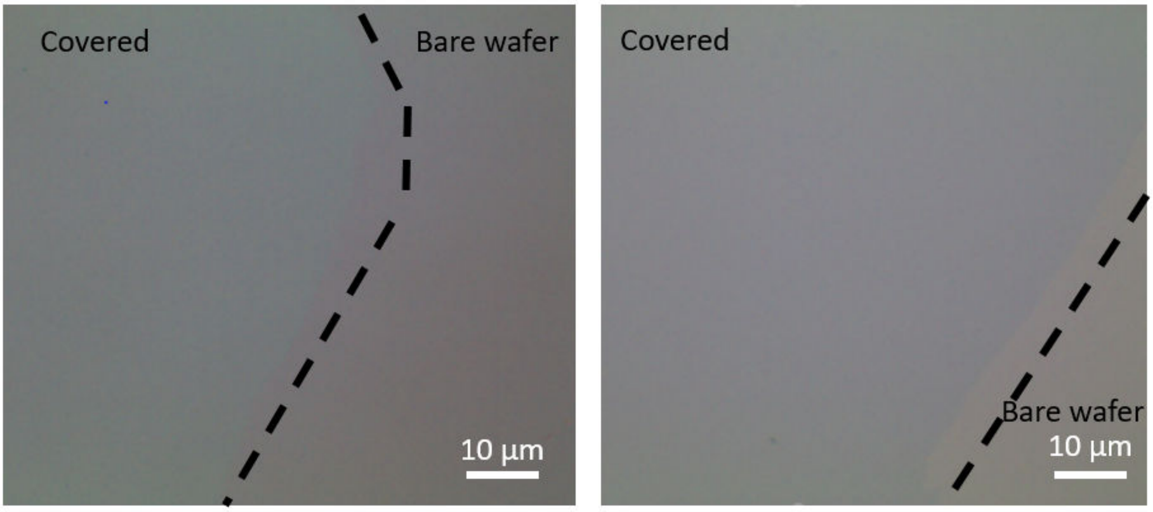

Fig. S8. Optical images of HTPHPB on Si wafer before (left) and after annealing at 1000 °C (right).

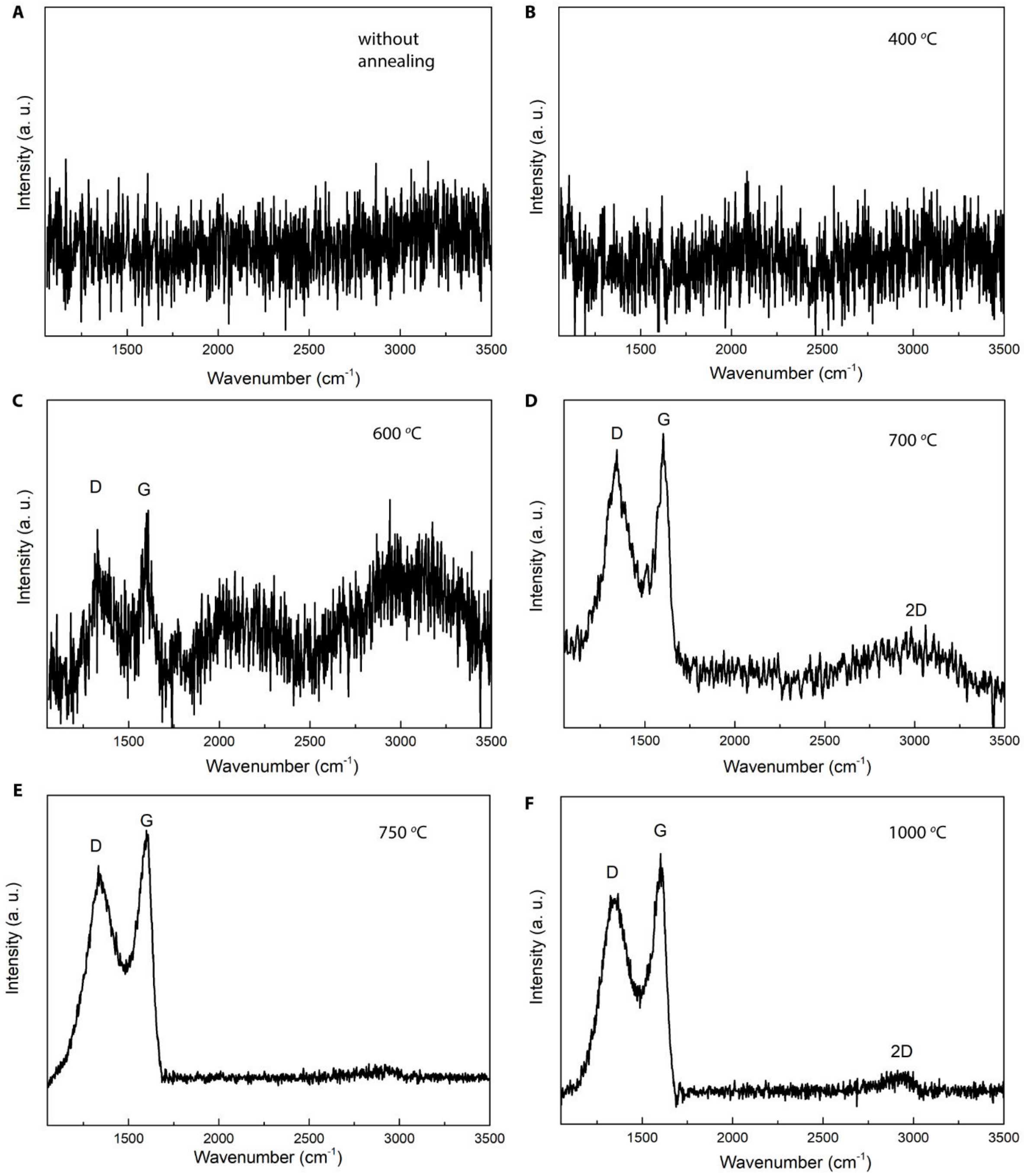

Fig. S9. Temperature-dependent Raman spectra of the monolayer without annealing and annealed at different temperatures.

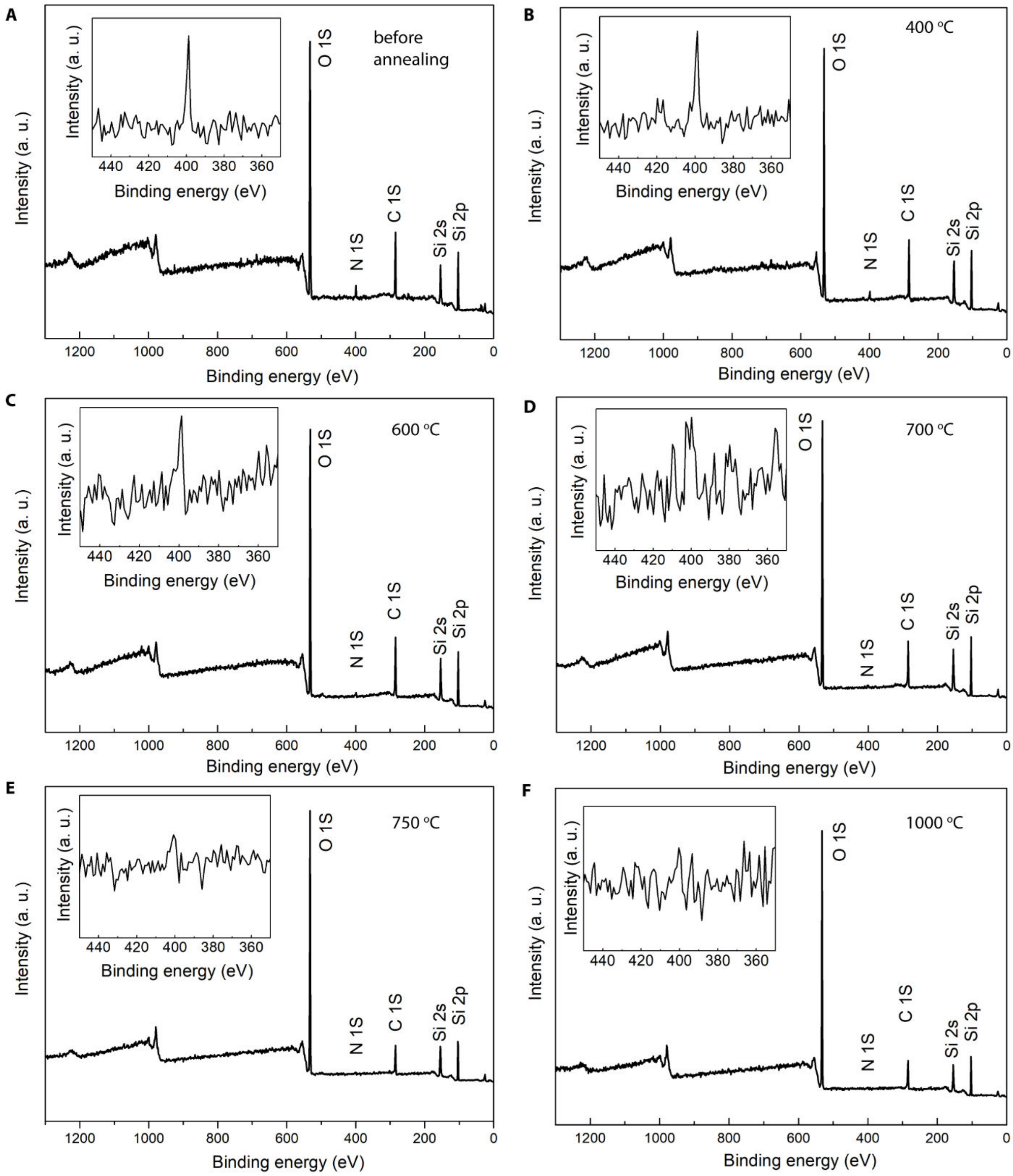

Fig. S10. Survey XPS of monolayer before annealing and annealed at different temperature.

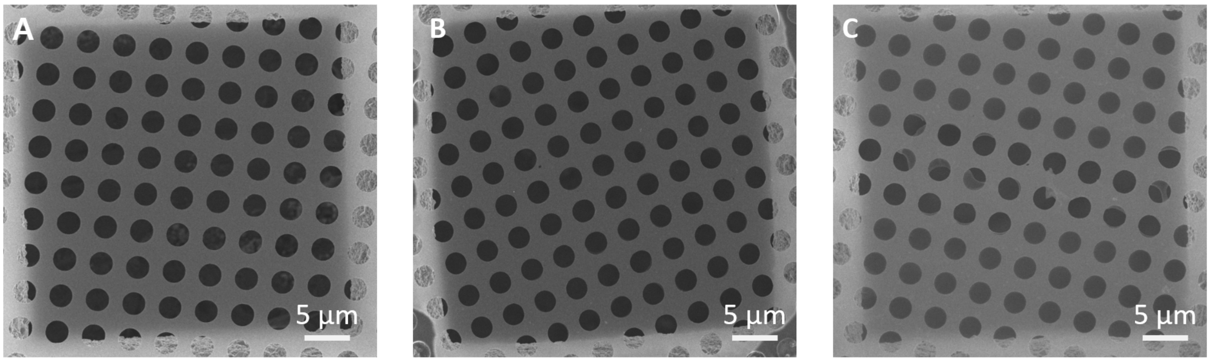

Fig S11. SEM images of monolayer annealed at (A) 400 °C (B) 600 °C and (C) 700 °C. The monolayer annealed at 700 °C is robust enough to be free-standing while the membrane prepared at 400 °C and 600 °C cannot be free-standing.

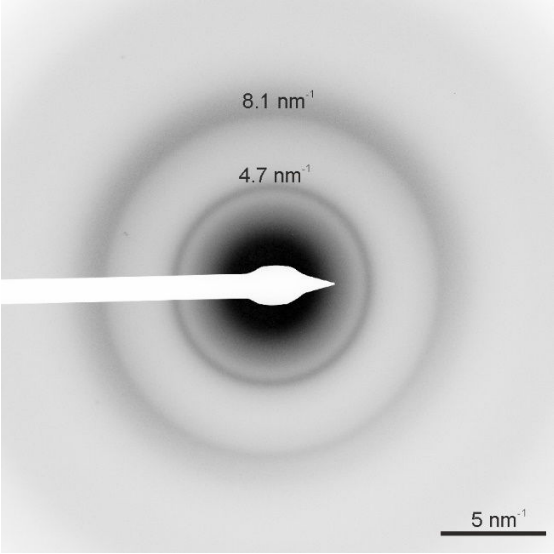

Fig. S12. Selected-area electron diffraction obtained from the nanocrystalline graphene membrane annealed at 1000 °C. The first and second diffraction rings are at 4.7 nm^{-1} and 8.1 nm^{-1}.

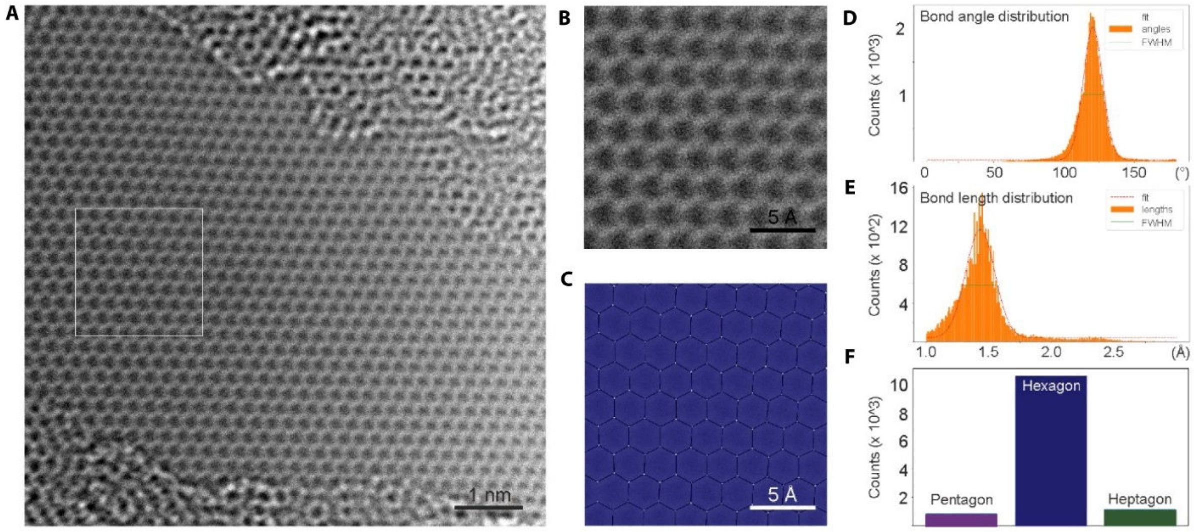

Fig. S13. AC-HRTEM characterization of CVD graphene monolayer. (A) AC-HRTEM image of the CVD graphene monolayer. Carbon atoms appear bright on a dark background. (B) Magnified image from the boxed region in (A). (C) Atomic positions and polygons mapped by neural network analysis. (D) Statistical analysis of bond angle distribution for CVD graphene monolayer (number of bond angles considered: 7×10^5). (E) Statistical analysis of bond angle distribution for CVD graphene (number of bond lengths considered: 4×10^5). (F) Frequency of polygons (number of polygons considered: 1.3×10^4). The polydispersity is raised from the image aberration and the machine learning algorithmic error.

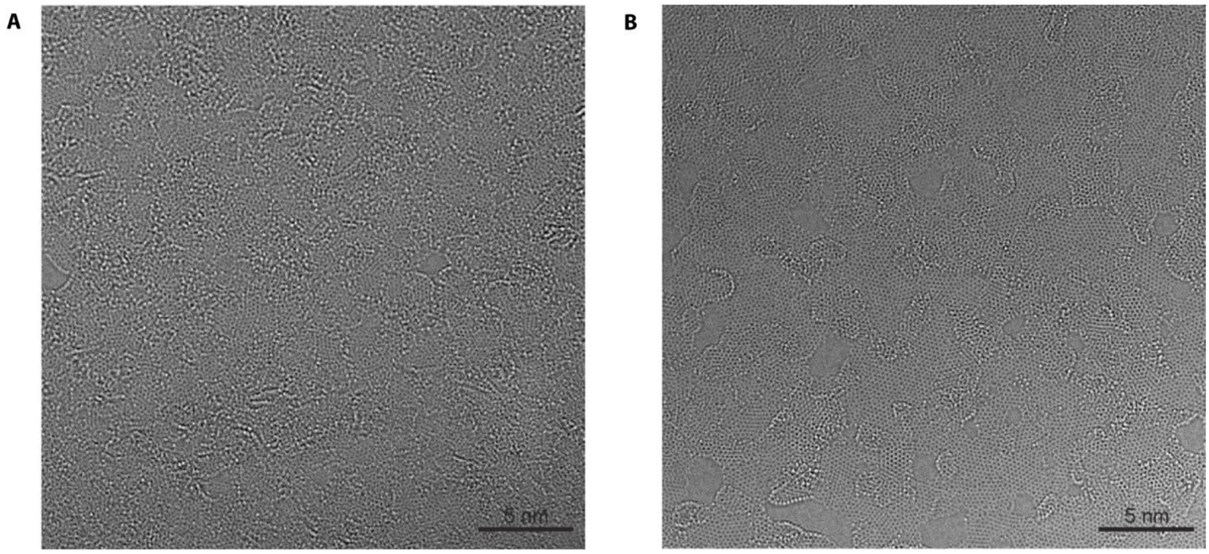

Fig. S14. Comparison between membranes annealed under 750 °C (A) and 1000 °C (B).

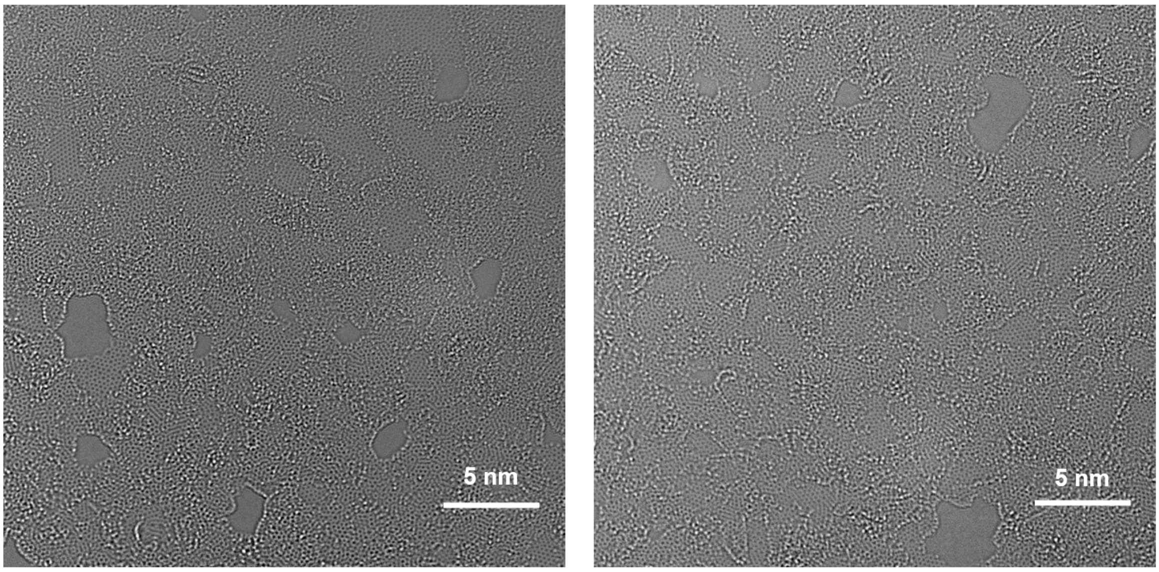

Fig S15. TEM images of nanocrystalline graphene annealed at 1000 °C.

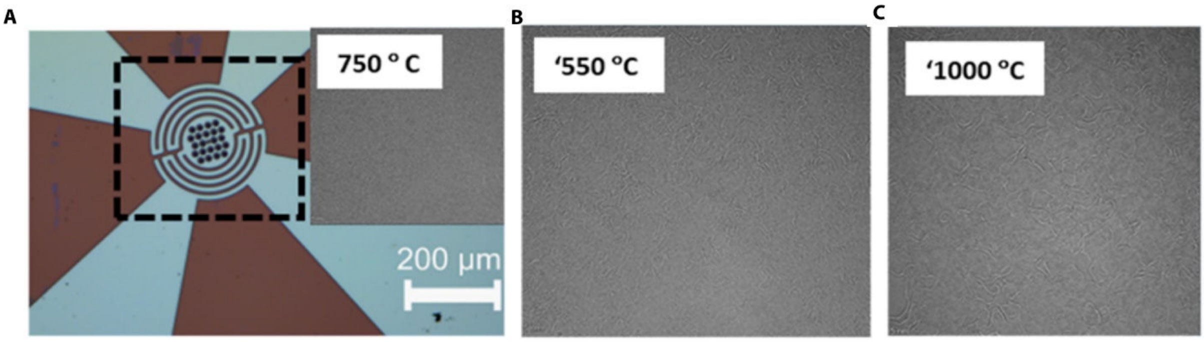

Fig. S16. HRTEM analysis of nanocrystalline graphene on MEMS chip at 750 °C after thermal crosslinking (A) and after secondary in situ annealing at 550 °C (B) and 1000 °C (C).

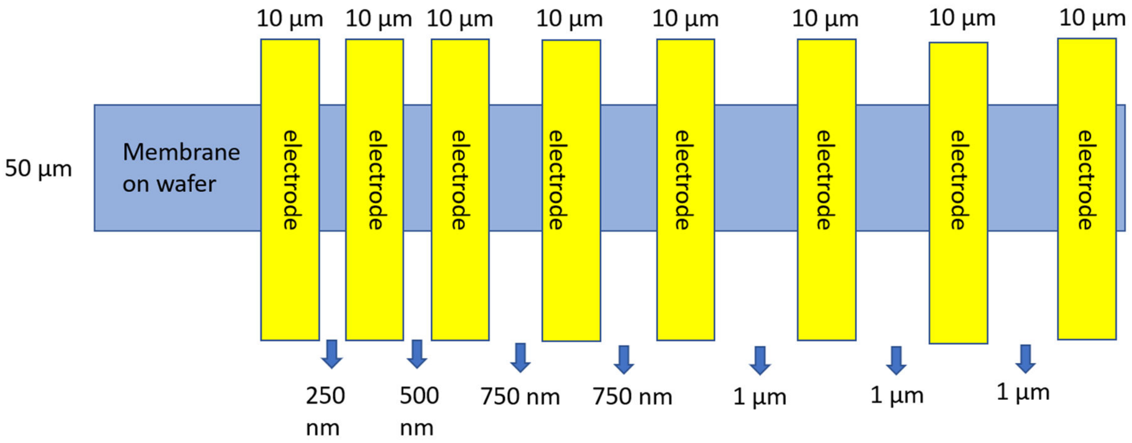

Fig. S17. The chip design for the conductivity measurements.

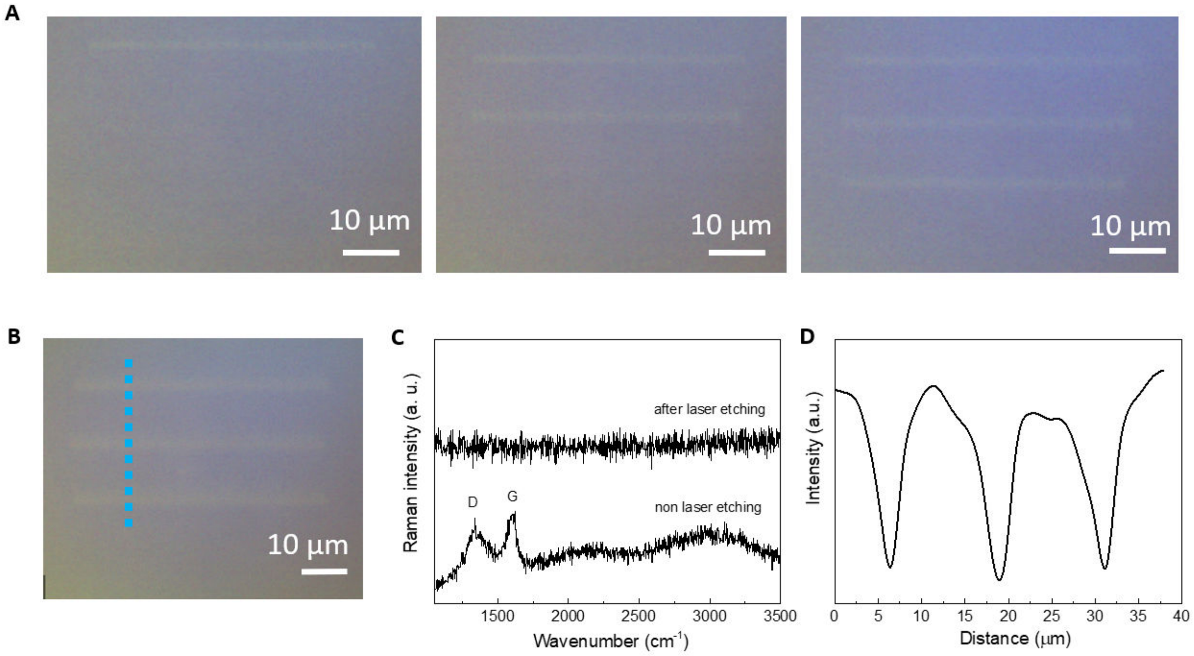

Fig. S18. Nanocrystalline graphene patterning with laser etching. (A) Optical images of real-time laser (457 nm) patterning on nanocrystalline graphene prepared at 1000 °C. (B) Line pattern on nanocrystalline graphene prepared at 750 °C. (C) Raman spectra of the laser-etched part and non-etched part in Fig. S18 A. (D) The G peak intensity monitored along the line in Supplementary Fig. S18 B.

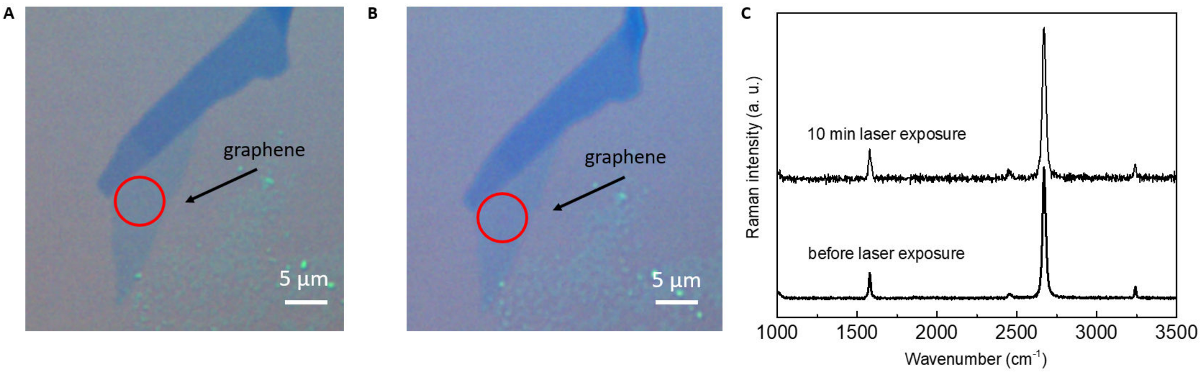

Fig. S19. Mechanical exfoliated graphene laser writing test. (A) Optical image of single layer graphene before laser exposure and (B) after laser exposure. (C) Raman spectra of mechanical exfoliated graphene before and after laser exposure. No clear changes can be observed in the spectra.

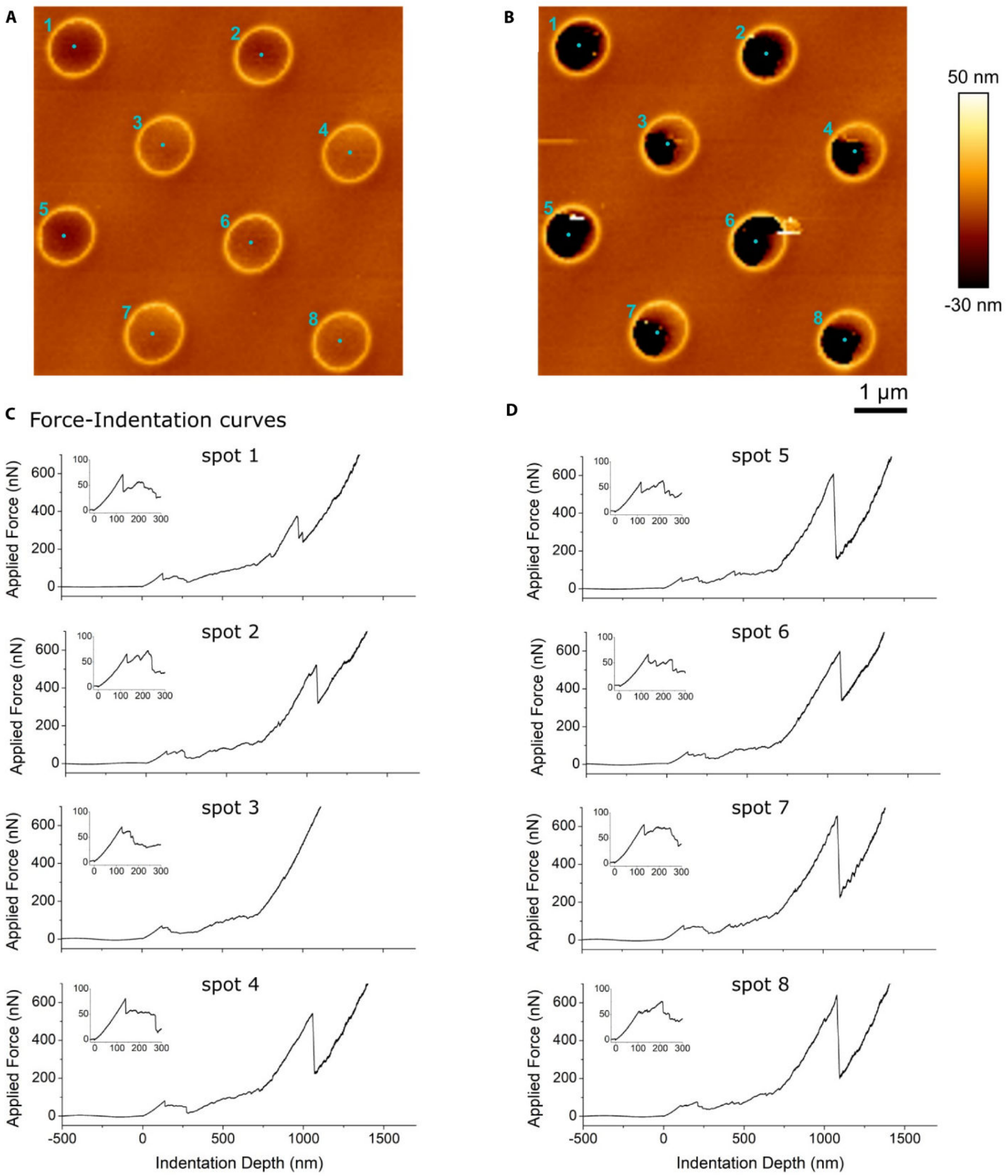

Fig S20. AFM point indentations of the nanocrystalline graphene. A and B AFM images of the nanocrystalline graphene covering the circular holes before (left) and after (right) the indentations. Blue dots show the points of indentation. C and D Force-Indentation curves from the spots depicted in the panel.

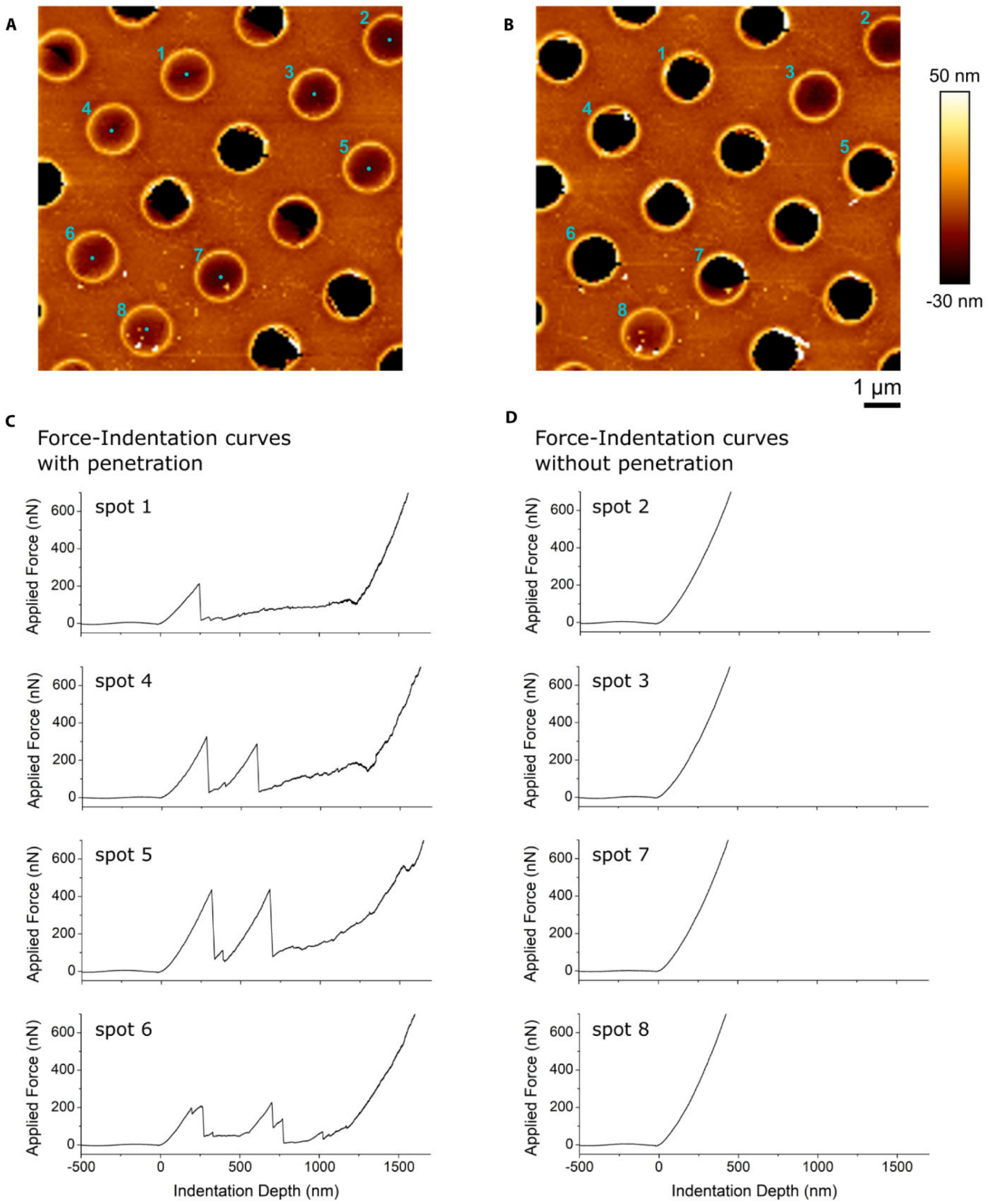

Fig. S21. AFM point indentations of the graphene film. A and B AFM images of the graphene film covering the circular holes before (left) and after (right) the indentations. Blue dots show the points of indentation. C and D Force-Indentation curves from the spots depicted in panel.

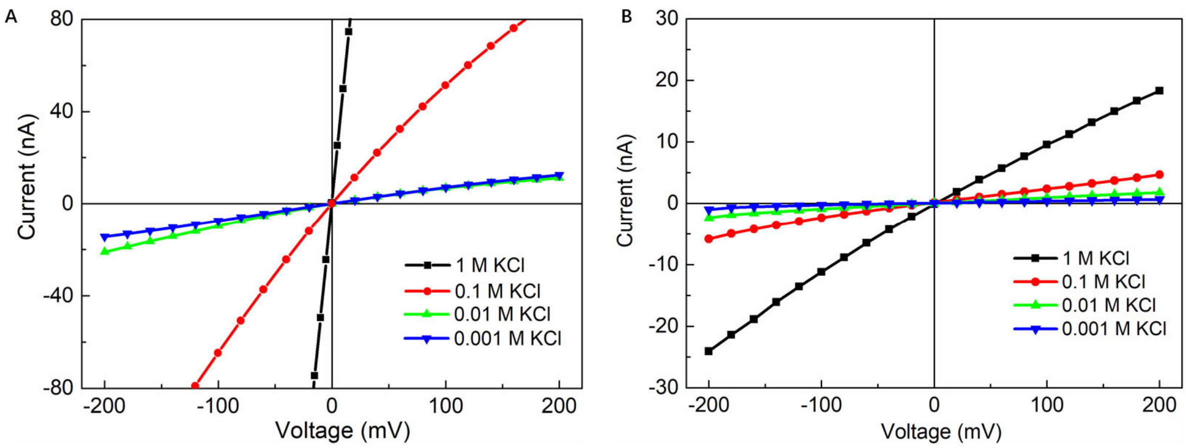

Fig. S22. The I-V curve of nanocrystalline graphene prepared at 750 degrees (A) and 1000 degrees (B) on SiN support.

The nanocrystalline graphene was transferred onto a SiN window with a 1 μm hole in the middle by PMMA method, and placed in a flow cell. Ag/AgCl electrodes were used to apply bias and the current was recorded by using Patch clamp (Axon 200B). For 1000 degrees sample, the conductance is about 91.5 nS in 1 M KCl. If the membrane surface contains only one nanopore, the pore diameter can be calculated using the equation below,

$$G = \sigma(\frac{4l}{\pi D^2} + \frac{1}{D})^{-1}$$

Where G is the conductance of the nanopore, σ is the conductivity of 1 M KCl solution, l is the thickness of the nanocrystalline graphene, and D is the pore diameter. The pore diameter calculated is 12 nm, corresponding to 113 nm^2 and one seven-thousandth of the freestanding membrane over 1 μm.

Table S1. DFTB-D3BJ single point energy results for 1-HTPHPB molecule and 5-HTPHPB molecules cluster in the first two rows. The third row shows the π-π stacking energy ($E_{5\text{-}HTPHPBcluster} - 5 \times E_{1\text{-}HTPHPB}$) resulting from the 5-HTPHPB cluster. The last row reports the π-π stacking energy for each of the 42 pyridines involved in π-π stacking interaction for the central X-shape core and external pairs of the 5-HTPHPB cluster. To obtain the last row value, the total pyridine π -π stacking interaction is divided by 42. The 42 pyridines are at an optimal π -π stacking average distance of ~3.6 Å.

**DFTB-D3BJ Single Point**	**Energy (kcal mol^{-1})**
5 x 1 HTPHPB	-941133.75
5 HTPHPB cluster	-941272.22
Total pyridine π-π stacking interaction	-138.47
Pyridine π-π stacking interaction for interacting pyridines	-3.30

Table S2. Statistical analysis of the polygon frequencies in nanocrystalline and CVD graphene (presented in percentage)

Sample	Pentagon	Hexagon	Heptagon
Nanocrystalline graphene	20%	69%	11%
CVD graphene	7%	84%	9%

Table S3. Assigned OPLS-AA force field atom types for HTPHPB generated by PRODRG server.

OPLS-AA atom types	Description
opls_145	Benzene C – 12 site JACS, 112, 4768-90.
opls_145B	Benzene C – 12 site JACS, 112, 4768-90, for biphenyl.
opls_146	Benzene H – 12 site.
opls_520	N in pyridine 6-31G*.
opls_521	C1 in pyridine CHELPG
opls_522	C2 in pyridine charges.
opls_523	C3 in pyridine.
opls_524	H1 in pyridine 520-619.
opls_525	H2 in pyridine.
opls_526	H3 in pyridine.
